# Psychometric Values of a New Scale: The Rett Syndrome Fear of Movement Scale (RSFMS)

**DOI:** 10.3390/diagnostics13132148

**Published:** 2023-06-23

**Authors:** Meir Lotan, Moti Zwilling, Alberto Romano

**Affiliations:** 1 Department of Physiotherapy, Ariel University, Ariel 4070000, Israel; 2 Israeli Rett Syndrome National Evaluation Team, Ramat Gan 5200100, Israel; 3 Department of Economics and Business Administration, Ariel University, Ariel 4070000, Israel; 4 Department of Health System Management, Ariel University, Ariel 4070000, Israel

**Keywords:** Rett syndrome, kinesiophobia, symptom assessment, evaluation tool, psychometrics

## Abstract

(1) Background: One of the characteristics associated with Rett syndrome (RTT) is a fear of movement (FOM). Despite the grave consequences on health, function, and the caregiver’s burden associated with bradykinesia accompanying FOM, there is no specific FOM assessment tool for RTT. (2) Objective: To construct and assess the psychometric values of a scale evaluating FOM in RTT (Rett syndrome fear of movement scale—RSFMS). (3) Methods: Twenty-five girls aged 5–33, including a research group (N = 12 individuals with RTT) and control group (N = 13 typically developing girls at equivalent ages). The Pain and Discomfort Scale (PADS) and Facial Action Coding System (FACS) assessed the participants’ behavior and facial expressions in rest and movement situations. (4) Results: Significant behavioral differences were recorded in these rest and movement situations within the research groups using the RSFMS (*p* = 0.003), FACS (*p* = 0.002) and PADS (*p* = 0.002). No differences in reactions were found within the control group. The new scale, RSFMS, was found to show a high inter- and intra-rater reliability (r = 0.993, *p* < 0.001; r = 0.958, *p* < 0.001; respectively), good internal consistency (α = 0.77), and high accuracy (94.4%). (5) Conclusions: The new scale for measuring FOM in RTT, the RSFMS, was validated using the FACS and PADS. The RSFMS was found to be a tool that holds excellent psychometric values. The new scale can help clinicians working with individuals with RTT to plan appropriate management strategies for this population.

## 1. Introduction

Rett syndrome (RTT) is a neurodevelopmental syndrome that affects about 7.1 per 100,000 females [1], making it the second most common multi-disability syndrome in females after Down syndrome [2]. Over 95% of individuals with RTT present a mutation in the methyl-CpG-binding protein 2 (MECP2) gene, which is located at the end of the long arm of the X chromosome at site Xq28 [3,4,5]. Despite the existence of a genetic diagnosis since 1999, the diagnosis of RTT is still based on clinical guidelines. These include normal pregnancy and delivery and a functional withdrawal that begins around one year, including a loss of acquired language, play, and motor skills, the appearance of stereotyped movements [6,7,8], and specific behaviors typical of that group of clients [9,10,11,12,13]. The behaviors that have been previously reported in the literature for RTT include mood changes [12], limited social interaction [10], self-injurious behaviors [13], stereotypical manual movements [14], and anxiety-like behaviors [10,13]. However, the behavioral characteristics of people with RTT are widely variable and complex relations between the behavioral phenotype and genetic mutation or clinical severity have been reported [10]. A few studies have described a high prevalence of behaviors such as anxiety, extreme mood swings, screaming events, and uncontrollable continuous crying in individuals with RTT [12,13,15,16,17,18,19,20]. It has been found that 87% of individuals with RTT present spells of apparent anxiety and fear in unfamiliar situations, some even at an extreme level [15]. Anxiety-like behaviors have also been detected in transgenic mice with RTT, indicating that this phenomenon is a core characteristic of RTT and should therefore be studied [21].

### 1.1. Fear of Movement

The concept of fear of movement (FOM) was first defined by Jane Ayers, an occupational therapist [22]. Ayers related this behavior to an under-sensitivity or hypersensitivity in the vestibular system and a poor ability of the central nervous system to effectively integrate sensory information from the limbic and vestibular systems and respond to movement challenges in a rapid and adapted manner. Therefore, the behavior of FOM was defined by Ayers as an impaired ability to present an adapted response against the force of gravity in certain situations [23]. According to Ayers, there are three levels of severity within the general term FOM: gravitational insecurity (the mildest level), intolerance to movements (the moderate level), and fear of movement (the extreme manifestation of this disorder).

FOM-like disorders cause the person who experiences them to avoid movement-associated actions that are perceived by the person with FOM as threatening, resulting in a decrease in functioning and participation or a significant delay in motor development in the case of children. Ayers recommended a treatment adapted to these three states of sensory sensitivity to movement [24].

The phenomenon of FOM in individuals with RTT was first introduced by Barbo Lindberg [25], a Swedish educator with experience in treating people with RTT. Lindberg believed, like Ayers before her, that FOM stems from a sensitivity of the vestibular system among these individuals. In contrast, a study by Lotan and associates [26] found that the FOM in RTT stems mainly from a difficulty in processing information from the proprioceptive system. This prevents those who suffer severely from FOM from enjoying any activity involving movement, such as walking, dancing, performing physical transitions, visiting playgrounds, and participating in activities in the community, and even makes it difficult for them to receive any form of external manipulation, such as the ones performed in physiotherapy treatments. Avoiding movement leads to a sedentary lifestyle, followed by a total lack of movement, which may lead to secondary disabilities such as joint deformities and significant muscle shortening, until becoming completely wheelchair-dependent [27]. This may affect the health and quality of life of people with RTT and their families. Hence, evaluating the phenomenon of FOM and providing appropriate treatment is necessary for this population to improve their quality of life and increase their involvement in various activities that, when controlled by FOM, are likely to be avoided. Furthermore, from a different point of view, FOM can be explained by the Fear Avoidance Model.

### 1.2. Fear Avoidance Model

The concept of FOM can be recognized and explained as part of a broader field of chronic musculoskeletal pain termed as the Fear Avoidance Model [28]. This model presents a sequence of causal factors, leading to a vicious cycle that links movement to chronic pain. The experience of an initial injury/damage/disability is perceived disproportionately (through catastrophizing thinking). This dispositional thinking is based upon and accompanied by incorrect and unobjective sensory processing, which may lead to fear being strongly associated with pain. This fear, in turn, leads to an excessive sensory arousal to pain and fear and the avoidance of movement (also termed kinesiophobia), which causes deconditioning and depression that perpetuates disability and promotes a more profound repetitive pain experience. This pathway may lead to a downward spiral vortex motion, gradually increasing this FOM. The FOM does not improve until the cycle of pain is broken and coping techniques are effectively implemented to disconnect the connection made by the person, integrating movement with pain until a complete or relative recovery. A recent literature review found strong evidence that FOM (kinesiophobia) is associated with high levels of pain and disability and moderate evidence for a positive correlation with decreased quality of life [29]. The review also found that FOM is a predictor of disability.

Although this model was originally intended to explain FOM for clients with an orthopedic background, it may also shed light on the phenomenon of FOM in RTT caused by the poor processing of information from the proprioceptive system, in addition to an inability to control the body resulting from difficulty in motor planning (apraxia) and difficulty in controlling movement (ataxia), all of which are characteristic of RTT [30].

The existing conventional measurement tools for FOM, such as the Fear Avoidance Beliefs Questionnaire and Tampa Scale for Kinesiophobia, are not sensitive enough and not individually adapted to the different levels of movement of different patients [31], as well as not adapted for individuals with RTT.

### 1.3. Evaluation and Measurement of Behavioral Characteristics in RTT

The current study deals with measuring and assessing the FOM among individuals with RTT. Due to their communicative limitations, self-reporting scales are not an option for those with RTT. Therefore, the next best option is to ask behavioral questions to their primary caregivers, as performed in the past in clinical studies among RTT populations [32]:

The Rett Syndrome Behavior Questionnaire (RSBQ) is designed to evaluate the behaviors among individuals diagnosed with RTT and includes a rating of various challenging behaviors [15].

Some of the limitations of the RSBQ are the lack of a direct evaluation of the person with RTT and the need to rely on reports from various direct care providers. In addition, this questionnaire is not specific to the aspect of FOM in RTT. As a result, the first author (ML), based on 34 years of clinical experience with this population, developed a new diagnostic tool that can be used for the specific assessment of FOM in individuals with RTT. The new assessment tool, The Rett syndrome fear of movement scale (RSFMS), has so far been validated by experts. The current research is a component of the development of this tool and deals with an evaluation of the additional psychometric characteristics of the tool: criterion validity, reliability, internal traceability, and sensitivity.

## 2. Methods

### 2.1. Ethical Issues

The research was approved by the Ethics Committee of Ariel University Institutional Review Board (AU-HEA-ML-20200918). Before the study initiation, all the participants’ parents signed an informed consent form for their daughters to participate in the study after receiving an explanation from the researchers about the research process. The neurotypical participants in the control group, aged 16 and above, signed their own informed consent forms.

### 2.2. General Description of the Study

The current study examined the characteristics of FOM in 12 girls with RTT compared to 13 age-matched participants showing normal development. The participants were videoed in resting state and movement situations (which will be described later). Three tests were carried out simultaneously for all the participants:

1. Testing FOM as observed by the participants’ body language using a new observational assessment tool (RSFMS) through video records.
2. Testing FOM as observed by the facial expressions of the participants by using the Facial Action Coding System (FACS).
3. Testing FOM as observed by the body language of the participants using the Pain and Discomfort Scale (PADS).

All the tests were carried out to validate the new observational assessment tool RSFMS in the form of receiving direct, objective feedback regarding changes in the body and facial reactions during Movement-free/Calm vs. movement situations of individuals with RTT. The evaluation was conducted by watching a video of the participants and their reactions to a minimal passive movement provided by an external facilitator (an experienced physiotherapist).

### 2.3. Research Hypotheses

1. Differences in facial and bodily reactions will be found between the calm and active situations in both groups.
2. In the movement situations, the facial expressions and body reactions will be found to be more pronounced among the participants with RTT (research group) compared to the control group, indicating a stress/anxiety/fear/excitement situation for the research group.
3. A highly significant and positive correlation will be found between the score of the new RSFMS assessment tool and the values of the behavioral/facial/bodily indicators, enabling the validation of the new tool.

### 2.4. Participants

The current study included a research group (N = 12) and control group (N = 13). The research group consisted of twelve girls aged 5–33 years who were genetically diagnosed with Classic RTT (according to their medical records at the National Rett Syndrome Specialist Clinic at Sheba Hospital, Ramat-Gan) and lived in the community (parents’ home). All the participants in the research group had previously been described in their medical records as showing FOM to various degrees as part of their behavioral and movement characteristics. A personal telephone call was made to all the relevant participants’ parents, even though their formal agreement was given, to confirm that they accepted having their child participate in the study. One family chose to withdraw from the study, leaving the research group with 12 participants (see Table 1).

The control group consisted of 13 females with typical development at matched ages to the research group. The researchers identified the participants in this group through personal acquaintances, while adjusting their age to the age of the participants in the study group.

### 2.5. Procedure

The physiological and behavioral responses to the movement of the participants in both groups were measured in an individual session at the participant’s home using all the measures (FACS and the PADS scales) in rest (Rest mode) and movement (Movement mode) situations. During the Rest mode, the participants sat on a comfortable chair or sofa for 10 min and watched a calm movie or listened to quiet music that was familiar to them, as chosen by their parents. The last five minutes were used to analyze their physiological/facial/behavioral reactions after a complete adaptation of all the participants to the position and after physiological relaxation had been achieved. Through the Movement mode, the participants sat on a stool. The same researcher (ML) executed all the movements of all the participants. Before activating this mode, what was expected to happen was explained to each participant, both verbally and through a short video that demonstrated the following order of movements:

- Movement sequence 1: the researcher moved the participant’s upper and lower limbs. For the upper limbs, the researcher held the participant’s wrists and performed five simultaneous and reciprocal arm movements up and downwards (flexion of both shoulders, one after the other) and five forward and back movements (flexion/extension of both elbows, one after the other). The lower limb movements were conducted by holding the participant’s ankles and performing five simultaneous and reciprocal lower limb movements up and down (flexion of both shoulders, one after the other).
- Movement sequence 2: the passive transfer of each participant from the seat she was sitting on to a nearby sofa/couch (20 cm away). The participant was lifted and moved by the researcher.

The participants were constantly videoed during the two measurements with a video camera that captured their entire body and facial expressions. This was performed to collect data, which were later analyzed to compare and evaluate their reactions using the new RSFMS measurement tool, the FACS, and the PADS. Two reviewers independently rated the participants’ reactions to measure the interrater reliability. One of the reviewers rated the responses of all the participants twice using a two-week interval to check the intra-rater reliability. Prior to the initial stages of the research protocol, the use of the RSFMS was taught by the tool developer (ML—tester 2) to the secondary researcher (tester 1). To teach the secondary researcher how to use the tool, the two researchers jointly filled in the tool sections on a video of an individual with RTT not participating in the current study. The two examiners were both physiotherapists working in the field of pediatrics. Moreover, the participant’s parents filled out the RSBQ behavior questionnaire during the measurement session.

### 2.6. Measurement and Evaluation Tools

#### 2.6.1. Rett Syndrome Behavioral Questionnaire (RSBQ)

The RSBQ, which was composed for individuals with RTT, is a measure that evaluates the frequency and intensity of different behaviors of a person with RTT. It was filled by a parent or direct care provider [15]. The scale is divided into eight subcategories/domains (general mood, breathing problems, body vibrations, manual movements, facial expressions, sleep problems, anxiety/fear, and walking and standing). Each item in the scale is scored 0–2 on the Likert scale. A score of 0 symbolizes a mismatch of the item for the specific participant, a score of 1 symbolizes a partial match, and a score of 2 signifies that the item most describes the individual’s behavior [15].

#### 2.6.2. Rett Syndrome Fear of Movement Scale (RSFMS)

The RSFMS (available as Supplementary Material Table S1: RSFMS data sheet) is a new tool based on the RSBQ. The new tool was developed and adapted specifically to behaviors clinically recognized as being associated with FOM in individuals with RTT, in accordance with the clinical experience of the first author (ML) with this population. Unlike the original RSBQ tool, the new tool contains only questions that deal with FOM in different situations and does not examine other behavioral aspects. Like the RSBQ, the rating is performed using a three-unit Likert scale (0–2), indicating how well each item describes the participant with RTT behaviors, as performed with the original tool (RSBQ).

#### 2.6.3. Facial Action Coding System (FACS)

The FACS was developed in 1976 by Paul Ekman and Wallace Friesen [33]. The process of developing this tool included isolating and mapping the facial muscles and reaching a total of 44 musculature movements of Action Units (AUs), which can be separated anatomically and visually discerned. The FACS has been used in over 100 studies in diverse populations (including people with intellectual disabilities). By using the FACS, a researcher can analyze a person’s facial expression to understand what emotions this person is experiencing. This scoring is performed by observing a person’s facial expression, breaking down the facial expression into the observed AUs and associating them with the emotion defined in the FACS dictionary, which defines which combinations of AUs make up a particular emotion. The psychological properties of the FACS have been found to be high in many versions of the scale: test–retest reliability (ICC = 0.928) and internal consistency (α = 0.880–0.904) were excellent [34].

#### 2.6.4. Pain and Discomfort Scale (PADS)

PADS is defined as a scale for assessing the pain and discomfort in individuals with intellectual and developmental disabilities (IDD). This scale is based on previous studies that have used facial expressions and body movements to indicate acute pain and discomfort, such as the Non-Communicating Children pain checklist [35]. PADS was designed to help medical professionals to identify, diagnose, and more effectively treat pain and discomfort in patients with severe communication problems. Validation studies by Bodfish and his associates [36] suggested that a convergent validity of PADS was achieved against the FACS, and very high intra and interrater reliabilities were reported (r = 0.93; r = 0.99, respectively) [37]. Other findings suggest similar results, attesting a high interrater reliability (ICC = 0.86–0.87), a sensitivity to pain behaviors in adults with IDDs (SRM = 0.52), and internal consistency (α = 0.74–0.78) [37]. Due to these findings, this tool has been used in studies on populations with IDD [38] and research including individuals with RTT [39].

### 2.7. Data Processing

The data were analyzed using SPSS version 25 software. Statistical significance was determined as *p* < 0.05. The analysis was performed in three stages. Initially, the averages and standard deviations of the study variables were calculated. The scores of the RSFMS and RSBQ were calculated as the sum of the items of the scales. The non-parametric Wilcoxon signed-rank test (equivalent to the paired samples *t*-test) was used to examine the differences in the physiological measures between the rest and movement modes in the research group and control group separately. The Mann–Whitney U test (the non-parametric alternative to the *t*-test in cases of small samples) was used to compare the study variables between the two groups. The variables’ scores were adopted from the literature and all the items were evaluated with an alpha Cronbach test, in order to make sure that they were consistent and that the alpha Cronbach was above. 0.7. Kendall’s Tau-b correlation coefficients were calculated to examine the relationship between the FACS, PADS, RSBQ, and RSFMS scores of the participants with RTT and α = 0.05 was set as the significance threshold for this analysis. Spearman correlations were conducted to assess the reliability (intra-rater and inter-rater reliability) characteristics of the RSFMS. In the current study, a-parametric tests were used, because the small sample size would not allow for parametric analyses with a reasonable intensity and because the dependent variable in the current study was not normally distributed according to the Kolmogorov–Smirnov test. The internal consistency of the questionnaire was tested using the Cronbach alpha index. The second stage focused on finding the cut-off point value for defining fear of movement using the new RSFMS assessment tool. For this goal, an ROC analysis test for bivalent classification was conducted. In the third stage, the true/false positive rates and false/positive negative rates of the two testers were examined separately in binary with a 2 × 2 table to examine the sensitivity and specificity of the tool, its predictive value, and its accuracy.

## 3. Results

This article presents the statistical analyses conducted to test the research hypotheses. The results section will be divided into several parts: descriptive statistics, the analysis of the physiological measures of the participants in the research group versus the control group, an examination of the relationship between the behavioral measures and the FACS, PADS, and RSFMS scores of the participants, and a presentation of the psychometric characteristics of the new RSFMS measurement tool.

### 3.1. Descriptive Statistics

Table 2 presents the averages and standard deviations of the study variables collected from the research and control groups. The statistical test did not find a significant difference in the age of the participants between the two groups (*p* > 0.05).

### 3.2. Validation Process

#### 3.2.1. RSFMS Scores

**Figure 1:**
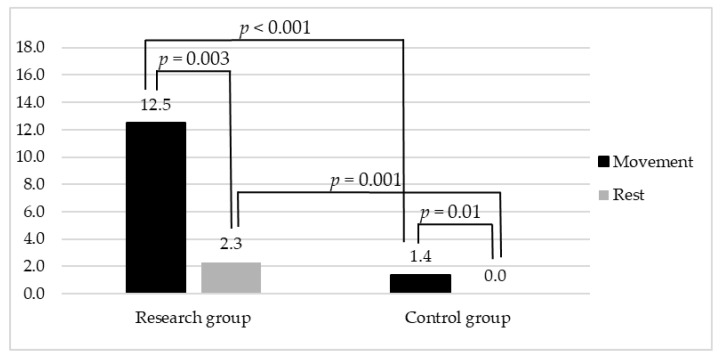
Graphical representation of data collected through RSFMS from research and control group within Rest and Movement modes.

The RSFMS scores significantly differed between the Rest and Movement modes among the research group (*p* = 0.003). A statistical difference emerged also between the Rest and Movement modes of the control group (*p* = 0.01), but the average score recorded with the RSFMS during the control group’s Movement mode was lower than the average score of the research group’s Rest mode score. Coherently, a significant difference was found when comparing the RSFMES scores collected from the research and control groups during the Movement (*p* < 0.001) and Rest modes (*p* = 0.001). The RSFMS data collected from the two groups are depicted in Figure 1.

#### 3.2.2. PADS

**Figure 2:**
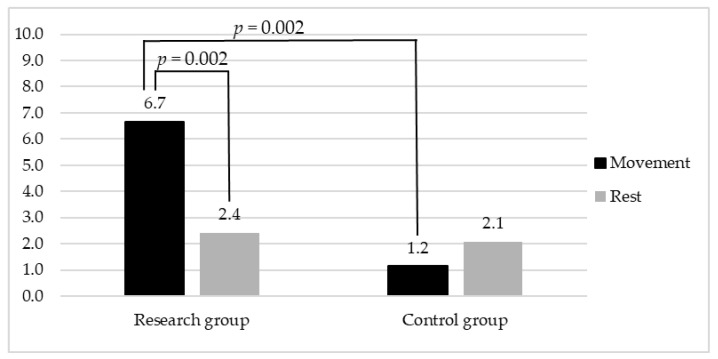
Graphical representation of data collected through PADS from research and control group within Rest and Movement modes.

The PADS scores significatively differed between the Rest and Movement modes among the research group (*p* = 0.002), but not among the control group (*p* = 0.19). Coherently, a significant difference was found when comparing the reactions to movement between the research and control groups (*p* ˂ 0.001). On the other hand, no difference was identified between the PADS scores collected from the two groups during the Rest mode (*p* = 0.44). The PADS data collected from the two groups are depicted in Figure 2.

#### 3.2.3. FACS

**Figure 3:**
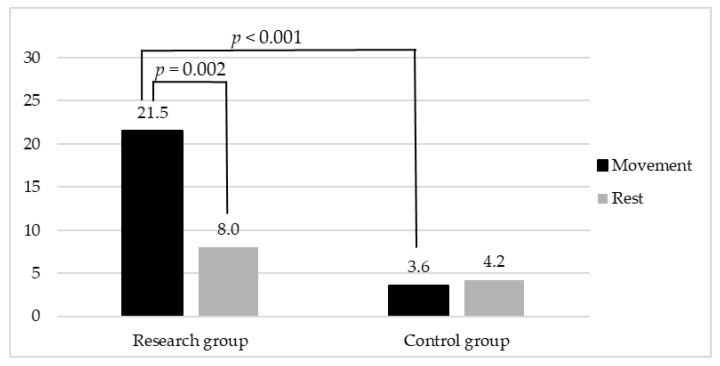
Graphical representation of data collected through FACS from research and control group within Rest and Movement modes.

A significant difference (*p* = 0.002) was identified between the Rest and Movement modes within the research group, while there were no statistical differences between the two modes in the control group (*p* = 0.103). Accordingly, the FACS data collected during the Movement mode significantly differed between the research and control groups (*p* ˂ 0.001), while no difference was found within the Rest mode. The FACS data collected from the research and control group are shown in Figure 3.

#### 3.2.4. Correlations

The Spearman’s rank correlation coefficient (rho) was measured and interpreted as follows: a value between 0.9 and 1.00 indicated a very strong correlation, between 0.7 and 0.89 indicated a strong correlation, between 0.5 and 0.69 indicated a moderate correlation, between 0.3 and 0.49 indicated a moderate to low correlation, between 0.16 and 0.29 indicated a weak to low correlation, and below 0.16 indicated a correlation that is considered too low to be meaningful [40,41]. Moreover, no statistical correlation was found between the RSFMS and PADS (*p* = 0.24, r = 0.281), FACS (*p* = 0.24, r = 0.260), and RSBQ (*p* = 0.25, r = −0.289).

#### 3.2.5. Psychometric Traits of RSFMS

The identified psychometric characteristics of the RSFMS are summarized in Table 2. The internal traceability of the new RSFMS assessment tool was evaluated according to Cronbach’s Alpha and ranged from 0.708 to 0.765, depending on the tester. It should be noted that it was possible to increase the internal traceability by reducing the items in the tool, yet reducing these items would have come at the expense of a loss in clinical sensitivity, and therefore it was decided to leave all the items.

In order to examine the inter-rater reliability and reliability between the different tests performed by the same examiner (intra-rater), Spearman correlations were calculated between the average RSFMS scores of the study group’s Movement modes between two testers and between two measurements of the same tester. A strong positive correlation was found between the evaluations of the different assessors (*p* < 0.001, r = 0.993) and between the different evaluations of the same tester (*p* < 0.001, r = 0.958).

#### 3.2.6. Cut-off Point for Defining Fear of Movement Using RSMFS

To find the cut-off point value for defining fear of movement using the new RSFMS assessment tool, an ROC analysis, a test for bivalent classification, was conducted. A cut-off point score of 0.18 was found and multiplied by the number of items in the RSFMS tool used to evaluate individuals with RTT. It is important to note that it is more accurate to use a cut-off point by averaging a score multiplied by a number of items rather than as a fixed score number, as not all the tool items are used in every assessment due to the heterogeneity of the functional level in girls with RTT. For example, a girl with RTT who does not walk is not rated with the RSFMS items that ask if anxiety is observed during walking. In this case, the number of items rated for this girl differs from another who does walk. Therefore, this cut-off point should be used as an average, as the score multiplies several items to achieve cut-off point uniformity.

#### 3.2.7. Sensitivity, Specificity, Positive/Negative Predictive Value, and Correctness

After the cut-off point value for fear of movement was defined in the new RSFMS assessment tool and a bivalent classification was made for “yes fear of movement” versus “no fear of movement”, the true/false positive rates and positive/false negative rates of the two testers were examined separately and binarily with a 2 × 2 table in order to test the sensitivity and accuracy of the tool.

The sensitivity value of the RSMFS (i.e., the percentage of girls with RTT who presented FOM identified as having FOM using the RSFMS) was 83.3% for tester 1 and 100% for assessor 2.

The specificity value of the RSMFS (i.e., the percentage of girls with RTT who did not present FOM identified as not having FOM using the RSFMS) was 75% for tester 1 and 83.3% for examiner 2.

The positive predictive value of the RSMFS (i.e., the percentage of girls with RTT who indeed presented FOM out of all the girls with RTT diagnosed as having FOM using the RSMFS) was 86.9% for tester 1 and 92.3% for tester 2.

The negative predictive value of the RSMFS (i.e., the percentage of girls with RTT who did not present FOM out of all the girls with RTT diagnosed as not having FOM by the RSMFS) was 69.2% for tester 1 and 100% for tester 2.

The RSFMS accuracy value (i.e., the rate at which the RSFMS correctly identified the presence or absence of FOM out of all the test results) was 85.5% for tester 1 and 94.4% for tester 2.

#### 3.2.8. Gaps in Tester Skill

According to the research array, tester 1 rated the views of the active situations of the control group twice, using the RSFMS to find the reliability between the tests, while tester 2 ranked them once to find the reliability between the testers. It should be noted that there was a discrepancy in the degree of experience that the two assessors had with using the RSMFS tool. This can be seen in the differences in the α (false positive) errors and β (false negative) errors of both testers. The percentage of α-type errors for tester 1 was 25% for the first and second measurements, while for tester 2, with extensive experience in using the RSFMS, the error rate was 16.6% (a gap of 66.4%). Accordingly, the percentage of β-type errors for the first measurement of tester 1 was 37.5% and decreased to 16% for the second measurement (a decrease of 42.6% in the percentage of error), while the β-type errors of tester 2 were at 0%.

## 4. Discussion

The current study was conducted with the aim of validating the new RSFMS assessment tool, which aims to evaluate the FOM in girls with RTT. The RSFMS was compared with the PADS and FACS. The results indicated that the study’s goal was achieved: the RSFMS was correlated with scales measuring similar concepts (content validity). Moreover, the new RSFMS assessment tool holds strong psychometric values of inter- and intra-rater reliability, internal traceability, and sensitivity.

### 4.1. Summary of Key Findings

This preliminary study involved 25 volunteer girls aged 5–33, 12 girls with RTT in the research group and 13 girls of equivalent ages presenting normative development. The findings suggest a significant difference between the reactions of both groups to rest and movement situations, yet this reaction was only found to be significant within the research group with both reference scales (PADS and FACS), as well as the new scale (RSFMS). Moreover, all three scales were able to distinguish between the calm and movement situations in the research group, but not in the control group, further validating both the RSFMS and the existence of FOM in the research population. However, the RSFMS score did not correlate with the FACS and PASD scores, suggesting that the facial and nonverbal expressions of pain and discomfort could not be reliable indicators of the presence of FOM. In addition, no correlation was found between the RSFMS and RSBQ questionnaire, indicating no relationship between the different content domains measured by the two different scales and that reporting general behavior does not predict the response of a person with RTT to movement. High attribute values were found in the psychometrics of the new RSFMF assessment tool, as detailed in Table 3.

### 4.2. The Nature and Level of the Movement in the Present Study

In the present study, the movement manipulations applied to the participants were slight passive movements of the four limbs and a transfer of the participant from chair to chair. The nature of the movement chosen was due to clinical considerations of the need to create a research uniformity between the motor levels of all the participants (with different postural and functional control), as well as an ethical consideration of choosing a relatively moderate movement, since this was a study on humans. However, it could be seen that even such a relatively moderate movement caused some physiological arousal and expressions of anxiety, as obtained in the presented analysis. However, it must be taken into account that there are different levels of movement and that the levels of anxiety in the girls with RTT varied according to the nature and level of the movement. For example, the slight passive movement of four limbs and the transition from a chair to chair are dissimilar to the movements included in a complete physiotherapy treatment (such as walking, going up and down stairs, and getting up from the floor), or therapeutic horseback riding or participating with the family in a challenging playground. As mentioned in the literature review concerning the Fear Avoidance Model, which is tangential to the field of the current research, one of its criticisms is that it is necessary to distinguish between the different levels of movement when talking about the avoidance or fear of them [28].

This division into different levels of movement is reflected in the World Health Organization’s International Classification of Functioning (ICF) model [42]. This model divides between the different levels of functioning of a person: from basic functioning at the level of “body functions” (concerning the physiological functions of the body’s systems), through to “activities” (the basic functioning of the person in themself without a connection with the environment, such as mobility or eating), and the high level of “participation” (the functioning of the individual in the family, social, and community environment to which they belong). Although the model deals with functioning, in the field of physiotherapy, it can also be used in the context of movement that is placed at the base of any functioning at any level. Hence, there is room to study and test the RSFMS also at higher levels of functional movement and classify the different levels of movement anxiety among people with RTT.

### 4.3. The Contribution of Research to the Clinical and Theoretical Fields

Since the FOM characteristic of many people with RTT causes their fixations when it is necessary to maintain free movement or when there is a need to deal with changes in position and movement dynamically, causing a reduction in their participation in movement experiences in addition, it is a hindering factor for normal motor development. Furthermore, FOM promotes a sedentary lifestyle and sometimes leads to unnecessarily needing the use of a wheelchair until a loss of walking ability in the individual with RTT. This is why it is necessary to address the component of fear of movement through its early diagnosis and adapted treatment.

The current research focused on the process of developing a diagnostic tool, the RSFMS, for FOM in girls with RTT. With this diagnostic tool, it is possible to identify FOM and track its changes throughout periodic treatment. The treatment of FOM includes, among other things, providing a sense of security by way of moving in safe and familiar situations, linking movement to positive experiences for the child by initiating mutual movement between the child and a familiar caregiver (parents, siblings, or care providers), gradually releasing defenses and fixations, and increasing the patient’s curiosity for various motor and sensory experiences [43].

### 4.4. The Limitations of the Study and Proposals for Further Research

As part of this study’s limitations, we can mention that this was a small study group with a wide age range, challenging the study external validity. Moreover, the small sample size prevented the use of the general recommendations provided in the COSMIN checklist for reporting measurement properties, which should be used in future investigations on the RSFMS’ psychometric characteristics. However, it should be noted that the participating girls were all the girls in Israel that have been reported over the past decade as suffering from significant FOM, based on the knowledge of the principal investigator (ML), who coordinates the physiotherapeutic treatment of this population in Israel, after reviewing all the reports he has written. In a follow-up study, it is recommended to try to sample a larger group of participants in the future by identifying more girls with RTT and FOM in Israel, or conducting the same follow-up study or a similar multifocal study in several other countries. Moreover, it is also recommended that the experimenters in a follow-up study undergo a more significant period of experience using the RSFMS. This suggestion is due to the significant decrease that was found in the degree of false positive and false negative errors between the first measurement of tester 1 (with no previous experience with a population of girls with RTT) and their second measurement, compared to the relative stability and high-accuracy percentages of the second tester (with about 30 years of experience with the study population) in identifying fear of movement.

Finally, conducting an in-depth study that will systematically examine the nature of the physiological responses of girls with RTT, with additional physiological measures such as ECGs, is recommended. In the present study, we observed, in retrospect, that some of the participants with RTT responded, physiologically, relatively late compared to the control group. Therefore, in the future, these physiological responses should be monitored for five additional minutes after the movement manipulation itself, and the degree of delay should be examined. In any case, whether or not a follow-up study will compare the RSFMS with physiological measures or FACS and PADS, there is room to perform more significant movement manipulations than those in the current study and examine the fear responses to different levels of movement.

## 5. Conclusions

The current study found that the RSFMS is a reliable tool with high internal traceability and sensitivity values. The scale was found to correlate with two other scales (PADS and FACS), which possess high psychometric values and have been used before in studies on individuals with RTT. Despite the fact that this scale needs further validation on larger numbers of participants with RTT, it is possible to rely on the psychometric values found for the tool and use it in clinics to diagnose FOM in girls and women with RTT.

## Figures and Tables

**Table 1:** Individual description of the research group.

N	Age	Ambulatory Status	Genetic Mutation
1.	8	Non-Ambulatory	c. 484C>T
2.	16	Assisted Ambulation	-----
3.	33	Non-Ambulatory	D156H
4.	20	Non-Ambulatory	R255X
5.	8	Assisted Ambulation	WT
6.	18	Assisted Ambulation	-------
7.	11	Non-Ambulatory	T158M
8.	10	Non-Ambulatory	C1157-1198 Del 41BP
9.	11	Assisted Ambulation	T158M
10.	19	Non-Ambulatory	62+1delGT
11.	18	Non-Ambulatory	R255X
12.	5	Independent Ambulation	R270X

**Table 2:** Descriptive statistics of the study variables.

		Age at the Study Beginning	RSBQ Scores	RSFMS Scores		PADS Scores		FACS Scores	
				Rest Mode	Movement Mode	Rest Mode	Movement Mode	Rest Mode	Movement Mode
Research group (N = 12)	Avg (SD)	14 (7.4)	38.8 (15.0)	2.3 (2.3)	12.5 (5.9)	2.4 (1.2)	6.7 (2.2)	8 (5.0)	21.5 (10.8)
	Min–Max	5–33	19–69	0–7	6.5–24.5	0–5	4–13	1–18	10–47
Control Group (N = 13)	Avg (SD)	12.8 (7.5)	-	0 (0.0)	1.4(1.4)	1.2 (1.2)	2.1 (2.5)	4.2 (2.1)	3.6 (2.2)
	Min–Max	5–33	-	0–0	0–4	0–7	0–4	2–8	1–8

Abbreviation list: RSBQ = Rett Syndrome Behavioral Questionnaire; RSFMS = Rett Syndrome Fear of Movement Scale; PADS = Pain and Discomfort Scale; FACS = Facial Action Coding System; Avg = Average; SD = Standard Deviation; Min = Minimum; and Max = Maximum.

**Table 3:** Summary of psychometric features of RSMFS.

Psychometric Feature of RSFMS	Value
Interrater reliability	r = 0.993
Intra-rater reliability	r = 0.958
Internal consistency	α = 0.765
Cut-off point	0.18 × Number of items on the scale used
Sensitivity	100%
Specificity	83.3%
Positive predictive value	92.3%
Negative predictive value	100%
Accuracy	94.4%

## Data Availability

The data supporting reported results can be found by the Corresponding author.

## Supplementary Materials

The following supporting information can be downloaded at: https://www.mdpi.com/article/10.3390/diagnostics13132148/s1, Table S1: RSFMS data sheets.
