# Supplementary material for: Psychometric Values of a New Scale: The Rett Syndrome Fear of Movement Scale (RSFMS)
Source: Diagnostics (Basel). 2023 Jun 23;13(13):2148. doi: 10.3390/diagnostics13132148 (PMC10502954; doi:10.3390/diagnostics13132148)
Supplement: Supplementary file 1 [file diagnostics-13-02148-s001.zip › diagnostics-2378700-SI.pdf]

Table S1: RSFMS data sheet

For each characteristic LISTED BELOW, please think about whether or not it accurately describes your daughter at this very point in time.

- For those characteristics that are VERY TRUE OR OFTEN TRUE please tick box 2.
- For those characteristics that are SOMEWHAT OR SOMETIMES TRUE please tick box 1.
- If the behaviour DOES NOT describe your daughter, please tick box 0 to indicate that the behaviour is NOT TRUE AS FAR AS YOU KNOW.
- If she is unable to perform any particular characteristic please also tick box 0.

Example

If your daughter uses gesturing very frequently to obtain desired objects, you would tick box 2 to indicate that it is very true or often true:

0	1	2 ✓

0	1	2	1. There are times when breathing is deep and fast (hyperventilation).
0	1	2	2. Spells of screaming hen body or libs are being moved during the day.
0	1	2	3. Makes extensive repetitive hand movements with hands when moved by others.
0	1	2	4. Occasions of breath holding when body or libs are being moved.
0	1	2	5. Air or saliva is expelled from mouth with force when hen body or libs are being moved by others.
0	1	2	6. Spells of apparent anxiety/fear in unfamiliar situations or when body or libs are being moved.
0	1	2	7. Seems frightened when there are sudden changes in own body position.
0	1	2	8. When body is moved by others parts of the body are held rigid.
0	1	2	9. Expressionless face when body or libs are being moved.
0	1	2	10. Spells of screaming when moved by others.
0	1	2	11. Abrupt changes in mood when body is moved.
0	1	2	12. There are times when she appears miserable when asked to sit unaided or stand walk with little support.
0	1	2	13. Screams hysterically for long periods of time and cannot be consoled when body or libs are being moved.
0	1	2	14. Although can stand independently tends to lean on objects or people. (*If your daughter does not stand please leave blank)*
0	1	2	15. Spells of laughter when body or libs are being moved during the day.
0	1	2	16. Makes mouth grimaces when body or libs are being moved.
0	1	2	17. There are times when she is irritable when asked to perform activities.
0	1	2	18. Spells of inconsolable crying when body or limbs are being moves during the day.
0	1	2	19. Makes grimacing expressions with face when body or libs are being moved.
0	1	2	20. Vocalises mostly shouts for dad and mom when body or libs are being moved.

0	1	2	21. Spells of laughter when body or libs are being moved.
0	1	2	22. Spells of apparent panic when body or libs are being moved.
0	1	2	23. Refuse to walk despite no apparent physical disability (spasticity, muscle shortening)
0	1	2	24. Walks with stiff legs despite no apparent physical disability (spasticity, muscle shortening – if not walking at all sign up as 2)
0	1	2	25. Spells of inconsolable crying when body or libs are being moved.
0	1	2	26. Closes eyes or turns head away (or other signs of rejection) when body or libs are being moved
0	1	2	27. Grinds teeth when body or libs are being moved
0	1	2	28. The child is unwilling to stand unaided and looks afraid/anxious when asked to do so
0	1	2	29. The child is unwilling to walk unaided and looks afraid/anxious when asked to do so
0	1	2	30. When the child is asked to walk/stand unaided she starts to hyperventilate and it takes her more than 1 minute to return to regular breathing
0	1	2	31. When the child is held on adult's hands and the adult is dancing without music the child looks afraid or shouts
0	1	2	32. When the child is standing and held at pelvic level by an adult, the child looks afraid or shouts
0	1	2	33. When the child is standing and held at shoulder's level by an adult, the child looks afraid or shouts
0	1	2	34. When the child is standing and held by her hands by an adult, the child looks afraid or shouts

**Sum scores**	
0-10	Normal
10-20	Mild fear of movement
20-35	Moderate fear of movement
35+	Severe fear of movement

(*Lotan, Zwilling, and Romano, 2023)*
